# Perceived cultural differences in healthcare for foreign patients visiting South Korea: tool development and measurement

**DOI:** 10.1186/s12913-019-3965-9

**Published:** 2019-03-28

**Authors:** Sumi Sung, Hyeoun-Ae Park

**Affiliations:** College of Nursing, Seoul National University, 103 Daehak-ro, Jongno-gu, Seoul, 03080 Republic of Korea

**Keywords:** Culturally competent healthcare, Nursing care, Medical tourism, Cultural differences, Tool development

## Abstract

**Background:**

We developed a 41-item tool measuring cultural differences in healthcare as perceived by foreign patients visiting South Korea.

**Methods:**

The tool was tested on 256 foreign patients who visited three tertiary hospitals in Seoul, South Korea. Content validity was explored by two physicians and eight nurses working in an international healthcare department. Structural validity was tested via exploratory factor analysis and by testing two hypotheses: (1) there are perceived cultural differences between the South Korean healthcare and those of foreign patients’ home countries (one-sample t-test); and, (2) Perceived cultural differences vary among language groups (analysis of variance). We also calculated Cronbach’s alpha.

**Results:**

The content validity index of the tool was 0.97. Exploratory factor analysis identified seven significant factors: hospital care and services, food, the healthcare system, communication, the healthcare facility, religion, and cultural values. The overall Cronbach’s alpha for the tool was 0.96, indicating very high internal consistency. We found that foreign patients visiting South Korean hospitals perceived that the healthcare culture differed significantly from that of their home country. The perceived cultural differences varied significantly by language group.

**Conclusions:**

Nurses can use our new tool to understand the cultural differences of foreign patients and provide them with culturally competent nursing care.

## Background

South Korea is becoming a multicultural society. Thanks to the Policy for Attracting and Utilizing Oversea Talents and Employment Permit System, the number of migrant workers has risen steadily [1]. In addition, South Korea attracts so-called medical tourists who travel for the purposes of medical treatment and healthcare [2]. Medical tourism has been promoted by some Asian countries [3] including South Korea, generally because high-quality healthcare is available at moderate cost. South Korea created a legal basis for attracting foreign patients by revising its medical laws in 2009, and began to promote medical tourism with the aim of increasing economic prosperity [4]. A report from the Korea Institute for Industrial Economics and Trade [5] ranked South Korea as the 19th most competitive country in medical tourism among the member nations of the Organization for Economic Cooperation and Development (OECD). In 2017, 321,000 foreign patients visited South Korea for medical reasons, which represents an annual average increase of 23.3% compared with 2009 [6]. In 2017, 31.0% of patients were from China, 13.8% from the USA, 8.5% from Japan, 7.7% from Russia, and 4.3% from Mongolia.

As the number of foreign patients visiting South Korean hospitals has increased, their cultural backgrounds have diversified. This rapid transition to cultural diversity within an ethnically homogeneous country has made many South Koreans, including healthcare professionals, uncomfortable [7]. Although the South Korean government and medical institutions have devoted much effort to creating a culturally friendly environment for foreign patients, this change in social demographics has been challenging for most South Korean nurses because they have rarely experienced, or been educated about, cultural diversity. The cultural competency of South Korean nurses is lower than that of nurses in the United States of America (USA) and Canada, whose societies have long histories of multiculturalism [8]. Moreover, foreign patients visiting South Korean hospitals experience difficulties associated with cultural differences in healthcare between South Korea and their home countries, in terms of the healthcare system itself, the food provided by hospitals, and language barriers [4, 9].

Patients treated in foreign countries expect to encounter cultural differences. However, if such differences are perceived negatively, this may cause patient dissatisfaction, poor adherence to medication, and worse health outcomes [10–12]. Thus, providing culturally competent nursing care to foreign patients requires that nurses first understand how patients view cultural similarities and differences [13], and then integrate patient values and preferences into their nursing care. As nurses become more knowledgeable about the cultural differences perceived by foreign patients, they will be able to understand the cultural beliefs of these patients and thus become better able to integrate patient values and preferences into their nursing care.

Previous studies in South Korea have considered only the nurses' point of views, measuring their cultural competence [8] and assessing their educational needs for cultural competence [14]. Few studies have explored whether foreign patients are satisfied with Korean healthcare services [15]. The available tools for measuring cultural issues in healthcare, such as the Cultural Self-Efficacy Scale [16], the Cultural Awareness Scale [17], the Cultural Competence Assessment [18], and the Cultural Knowledge Scale [19] measure only the cultural competence of nurses. No previous study has assessed how foreign patients perceive cultural differences in healthcare. This renders it impossible for nurses to understand how foreign patients experience differences in healthcare services, and the extent of such differences. In this study, we developed a tool measuring cultural differences perceived by foreign patients visiting South Korean hospitals.

## Methods

### Study design

We developed a tool measuring cultural differences in healthcare perceived by foreign patients visiting South Korean hospitals. The study was divided into two phases: (1) tool development of perceived cultural differences in healthcare by foreign patients; and (2) measurement of perceived cultural differences in healthcare by foreign patients.

The first phase involved defining various domains of cultural differences in healthcare perceived by foreign patients; generating the items comprising the tool; evaluating content validity; performing two rounds of stakeholder feedback via face-to-face interviews and a pilot study based on the “Core Outcome Measures in Effectiveness Trials” (COMET) handbook, version 1.0 [20]. In the second phase, we measured healthcare cultural differences perceived by foreign patients, and evaluated reliability and validity.

### Phase 1: Development of a tool measuring cultural differences in healthcare perceived by foreign patients

#### Defining the scope of perceived cultural differences in healthcare

We first reviewed the literature to define perceived cultural differences in healthcare. Leininger and McFarland [21] stated that: “Culture is the values, beliefs, norms, and practices of a particular group that are learned and shared and that guide thinking, decision, and actions in a patterned way.” Based on this definition, cultural differences is defined as differences in group values, beliefs, norms, and practices that are learned and shared, and that guide thinking, decisions, and actions. As we were concerned with cultural differences in healthcare perceived by foreign patients, we designed a tool by which patients can compare their own culture with South Korean culture in a set of domains reflecting various aspects of healthcare.

Before generating the items of the tool, we identified the domains of cultural differences relevant to healthcare from literature review. Communication, food, and religion domains were extracted from the six phenomena comprising the Transcultural Assessment Model of Giger and Davidhizar [22] and the 12 cultural domains of the Purnell Model for Cultural Competence [23]. The six phenomena of the Giger and Davidhizar include communication, space, social orientation, time, environmental control, and biological variation. The 12 domains of the Purnell model include overview/heritage, communication, family roles and organization, workforce issues, biocultural ecology, high-risk behavior, nutrition, pregnancy, death rituals, spirituality, healthcare practices, and healthcare providers. The communication domain was included in both models. The religion domain was included as the social orientation domain of Giger and Davidhizar and as the spirituality domain of the Purnell model. The food domain was included as the biological variation phenomenon of Giger and Davidhizar and as the nutrition domain of the Purnell model. The communication domain includes both verbal and non-verbal communication; the food domain includes the quality of food provided and the extent to which staff understand the patient’s food culture; the religion domain includes the available religious facilities and the extent to which staff understand the patient’s religion.

The following four domains were extracted from Flores [24] and Lynn and Deanna [25]: healthcare facility, health beliefs, patient-caregiver relationship, and healthcare system. The healthcare facility domain includes environmental features, such as the layout of the hospital room; the health beliefs domain includes traditional beliefs shaped by specific cultural beliefs; the patient-caregiver relationship domain includes interactions between patients and healthcare providers; and the healthcare system domain includes the mode of referral and the number of nurses per patient.

#### Determining what to measure

We generated items based on the literature review reflecting the values, beliefs, norms, and practices of particular groups that are learned and shared, and guide thinking, decisions, and actions. The items for the food and patient-caregiver relationship domains were developed by reference to two earlier tools: the Customer Satisfaction Survey for Nutrition and Food Service and the Patient-Doctor Depth of Relationship scale [26, 27]. Items for the health beliefs domain were developed by reviewing the literature on health beliefs by cultural background [28]. Items for the communication domain were developed by reviewing the literature on language barriers in healthcare [29, 30]. Items for other domains, including the healthcare system, the healthcare facility, and religion were developed by reference to Giger [31] and Purnell [23]. In total, seven cultural domains comprising 37 items were developed: 3 items on religion, 6 items on communication, 4 items on the healthcare facility, 7 items on food, 4 items on health beliefs, 5 items on the patient-caregiver relationship, and 8 items on the healthcare system.

#### Stakeholder feedback round 1: Face-to-face interviews

Face-to-face interviews were conducted with four medical coordinators and six patients to validate the seven identified cultural domains and generate additional items. A convenience sample of four senior medical coordinators working at one of the study hospitals were recruited. They were a Chinese-speaking coordinator from South Korea, a Mongolian-speaking coordinator from Mongolia, a Russian-speaking coordinator from Kyrgyzstan, and an Arabic-speaking coordinator from South Korea. All had worked for more than 3 years as medical coordinators in one of our study hospitals. Six patients were recruited by the snowball technique via the English, Chinese, and Arabic language coordinators. The six patients included two Arabic-speaking patients from the United Arab Emirates (UAE), two English-speaking patients from the USA, and two Chinese-speaking patients from China. These six patients represent the English, Chinese, and Arabic language groups which native medical coordinator were not represented. The first author explained the purpose of the study and conducted semi-structured interviews with four medical coordinators and six patients, lasting 20–40 min. They were asked to review the seven identified cultural domains and state whether they had experienced any other cultural differences in healthcare between South Korea and their home countries.

#### Translation

Four bilingual professional translators translated the English tool into Arabic, Russian, Chinese, and Mongolian. Another four bilingual translators performed back-translation into English. Eight translators reviewed the four translated and back-translated tools. We chose these five languages because most foreign patients treated in South Korea came from China, Russia, the USA, Kazakhstan, the UAE, and Mongolia. According to Jeanrie and Bertrand [32], and Peña [33], linguistic and cultural equivalence were assured by back-translation involving two native translators bilingual in English and one of the four other languages who understand the different cultural backgrounds.

#### Content validity

Content validity was evaluated by two physicians and eight nurses working in the international healthcare department at one of our study hospitals. The first author emailed 10 experts explaining the purpose of the study. All subjects were not involved either in validating the seven identified cultural domains or generating the items for the tool. The first author distributed questionnaires to those who agreed to participate. They were asked to rate the relevance of the 41 items of the tool to the seven domains of cultural differences using the 4-point scale: 1 = not relevant, 2 = somewhat relevant, 3 = quite relevant, and 4 = highly relevant. Completed questionnaires were placed in a dropbox located in the international healthcare department where the respondents worked.

#### Stakeholder feedback round 2: Pilot study

We performed a pilot study to evaluate whether all items were readily understood, and the time required to complete the questionnaire. We selected 20 foreign patients using a convenience sampling who visited one of the study hospitals. The first author explained the purpose of the pilot study to five medical coordinators working in one of our study hospital. These coordinators recruited six English-, five Arabic-, three Chinese-, three Russian-, and three Mongolian-speaking patients who visited the study hospital from May 29, 2016 to June 2, 2016. The first version of the questionnaires was distributed to the 20 foreign patients who agreed to participate. The medical coordinators recorded the time required to complete the questionnaire and asked the respondents how well they understood each item.

### Phase 2: Measurement of cultural differences in healthcare perceived by foreign patients

#### Participants

We enrolled 256 foreign patients who visited three tertiary hospitals in Seoul, South Korea. The enrolment criteria were age over 19 years; the ability to read and understand the questionnaire; the ability to communicate in English, Arabic, Mongolian, Chinese, or Russian; an understanding of the purpose of the study; and agreement to participate. The minimum sample size required to assess validity and reliability was calculated to be 205, corresponding to five times the total number of items [34].

#### Data collection

Data were collected over 3 months from August to October 2016 in three hospitals in Seoul that agreed to participate following institutional review board approval (IRB no. 1606–121-772). As the respondents were not South Koreans, the first author introduced the research to 15 medical coordinators (1 for each language at each hospital). The first author and the medical coordinators explained the purpose of the study to prospective respondents as they were leaving the hospital, and distributed questionnaires to those who agreed to participate. We asked the respondents to place completed questionnaires in dropboxes located in the international healthcare departments of the study hospitals. In total, 260 questionnaires were returned, of which 256 had been completed.

#### Quality evaluation

We evaluated the validity of the tool by measuring structural validity and testing hypotheses, and the reliability of the tool by measuring internal consistency. Based on the recommendation by Gorsuch [35], we used exploratory factor analysis (EFA) to explore structural validity because the tool was not based on a model or theory related to cultural differences in healthcare. Validity was further evaluated by testing two hypotheses. Foreign patients visiting South Korea for medical treatment will almost certainly perceive cultural differences. One useful validation procedure involves determining whether cultural differences perceived by foreign patients (compared to their native countries) differ significantly from 0, which corresponds to no difference. Hypothesis 1 was that the cultural difference in healthcare perceived by foreign patients is not equal to zero. Kramsch and Widdowson [36] state that culture is expressed, embodied, and symbolized by language. Therefore, the cultural differences in healthcare perceived by foreign patients will vary by their language. We thus also explored whether the cultural difference in healthcare perceived by foreign patients visiting South Korea differed by language. Hypothesis 2 was that cultural differences in healthcare perceived by foreign patients would differ by language group.

### Statistical analysis

Data were analyzed using SPSS software (ver. 21.0; SPSS Inc., Chicago, IL, USA). We performed a descriptive analysis to identify general characteristics of the respondents. We evaluated the structural validity and internal consistency of all items with mean, standard deviation, skewness, kurtosis, and corrected item-total correlation values. Structural validity was analyzed via EFA using varimax rotation and Kaiser normalization. We considered that structural validity was evident when the Kaiser–Meyer–Olkin (KMO) parameter was ≥0.80 and the Bartlett test of sphericity yielded a *p*-value < 0.05. We determined the number of factors to retain using eigenvalue-greater-than-1 rule, and eliminated items exhibiting factor loadings of < 0.40 or > 0.95. We labeled all extracted factors based on the cultural domains into which they fell during development, and on literature reviews on healthcare factors influencing patient satisfaction [37, 38].

Hypothesis 1 (that the cultural difference in healthcare perceived by foreign patients is not equal to zero) was tested using the one-sample *t*-test. Hypothesis 2 (that cultural differences in healthcare perceived by foreign patients would differ by language group) was tested employing analysis of variance (ANOVA). We converted the mean scores for all factors to percentages, and compared these across all language groups. We used Cronbach’s alpha as a measure of internal consistency.

## Results

### Phase 1. Development of a tool measuring cultural differences tool in healthcare perceived by foreign patients

#### Stakeholder feedback round 1: Face-to-face interviews

We found that the seven cultural domains required no major adjustment. However, four additional items were suggested for the health beliefs and healthcare facility domains. The Chinese-speaking medical coordinator suggested that a “focus on Western medicine in healthcare service” should be incorporated into the health beliefs domain, and the two Arabic-speaking patients suggested that “gender difference between patient and medical staff” should be included. In terms of the healthcare facility domain, the two English-speaking patients suggested that “common areas in the hospital” and “privacy in the hospital room” should be covered.

#### Validity: Content validity

The content validity index (CVI) was 0.97 for the overall tool and > 0.8 for each item. Two items were revised based on their CVI values. The item “gender difference between patient and main caregiver” was changed to “gender difference between patient and medical staff” because the word “caregiver” was unfamiliar to foreign patients. We added an explanation to the item “focus on Western medicine in healthcare”; the new item contained the phrase: “Western (not traditional) medicine”, to help patients understand the item better.

#### Stakeholder feedback round 2: Pilot study

The pilot study participants understood each item well, and took 10–15 min to complete the questionnaire. No major revision was required.

#### Final version of the tool

The final tool included 41 items that passed two rounds of stakeholder feedback and the content validity test: three items on religion, six items on communication, six items on the healthcare facility, seven items on food, six items on health beliefs, five items on the patient-caregiver relationship, and eight items on the healthcare system. Each item was scored using the 5-point Likert scale: extremely different = 4, very different = 3, quite different = 2, not very different = 1, and not different at all = 0. A higher total score indicates that the patient perceives greater cultural differences in healthcare between his/her native country and South Korea.

### Phase 2: Measurement of perceived cultural differences in healthcare perceived by foreign patients

#### General characteristics of the participants

In total, 256 respondents in three hospitals completed the questionnaire. Their primary languages were Arabic (*n* = 70, 28.5%), Russian (*n* = 60, 23.4%), Mongolian (*n* = 60, 23.4%), Chinese (*n* = 40, 15.6%), and English (*n* = 23, 8.9%). Most were female (*n* = 144, 56.2%), had been hospitalized previously (*n* = 135, 52.7%), were educated to college level or higher (*n* = 188, 73.4%), and were paying their medical costs themselves (*n* = 154, 60.2%). The largest religious group was Muslim (*n* = 97, 37.9%). An overview of the characteristics of the respondents is presented in Table 1.

**Table 1:** Characteristics of the Participants (*N* = 256)

Characteristics	n (%)
Language	
English	23 (8.9)
Chinese	40 (15.6)
Arabic	73 (28.5)
Russian	60 (23.4)
Mongolian	60 (23.4)
Sex	
Male	112 (43.8)
Female	144 (56.2)
Religion	
Christian	42 (16.4)
Buddhist	54 (21.1)
Muslim	97 (37.9)
Other	39 (15.2)
None	24 (9.3)
Education level	
Elementary school or lower	13 (5.1)
Middle school	8 (3.1)
High school	47 (18.4)
College	94 (36.7)
Higher than college	94 (36.7)
Type of visit	
Inpatient	135 (52.7)
Outpatient	121 (47.3)
Payment	
Self	154 (60.2)
Private insurance company	12 (4.7)
Korean National Health Insurance	7 (2.7)
Government	73 (28.5)
Other	10 (3.9)
Length of stay in South Korea	
Up to 1 week	46 (18.0)
More than 1 week and up to 1 month	84 (32.8)
More than 1 month and up to 6 months	64 (25.0)
More than 6 months and up to 1 year	17 (6.6)
More than 1 year	45 (17.6)

#### Validity: Structural validity

For each item, we calculated the mean, standard deviation, skewness, and kurtosis. The mean total score for all items was 59.73 ± 28.17 (mean ± SD) and no item exhibited a deviation (skewness or kurtosis) > 2.0. The corrected item-total correlation coefficient ranged from 0.67 to 0.90; thus, no item was removed. We applied the KMO test and Bartlett’s test of sphericity before performing EFA; these revealed that sampling adequacy was high (KMO measure = 0.93; *p* < 0.001). Thus, all 41 items were entered into the EFA, which identified the seven factors shown in Table 2 based on the EFA eigenvalues with no item being eliminated. The factor loading for all items was > 0.40, ranging from 0.450 (“cost of medical care”) to 0.925 (“food temperature”). The overall factor structure accounted for 73.70% of the cumulative variance.

**Table 2:** Factor Loadings of the 41 Items (*N* = 256)

Items	Factor number						
	1	2	3	4	5	6	7
6. Empathy shown by medical staff	.83						
21. Convenient facilities in the hospital (e.g., bank, convenience store, café)	.76						
22. Sanitary facilities in the hospital (e.g., toilets, baths, showers)	.74						
23. Health and illness perception	.73						
24. Attitude of medical staff toward my illness	.72						
27. Informing the patient about the diagnosis or prognosis	.70						
29. Caring time spent by medical staff	.69						
30. Caring attitude of medical staff	.69						
31. Understandability of information given by medical staff	.58						
32. Friendliness of medical staff	.53						
33. Skill and competency of medical staff	.52						
34. Waiting time for consultation	.50						
41. Cost of medical care	.45						
10. Food temperature		.93					
11. Food appearance		.92					
12. Food taste		.92					
13. Food aroma		.88					
14. Food service		.84					
15. Attitude of medical staff toward my food preferences		.79					
16. General awareness of food taboos predicated by culture/religion		.68					
35. System of referral from a primary-care clinic to a tertiary hospital			.70				
36. Protecting privacy while providing healthcare service			.69				
37. Involvement of family members in caring for patient			.68				
38. Hand hygiene of medical staff (e.g., not wearing medical gloves when providing medical services)			.68				
39. Number of nurses caring for me			.65				
40. Globalization of healthcare service			.64				
1. Spoken communication with medical staff				.79			
2. Medical terms used by medical staff (e.g., brand names of medications)				.74			
3. Gestures of medical staff				.73			
4. Physical contact by medical staff				.71			
5. Eye contact of medical staff				.68			
17. Sharing the hospital room with other patients					.79		
18. Privacy in the hospital room					.75		
19. Layout of the hospital room					.71		
20. Common areas in the hospital (e.g., kitchen, waiting room)					.62		
7. Religious facilities in the hospital (e.g., church, prayer room)						.85	
8. Religious ceremonies offered at the hospital (e.g., mass, prayers)						.83	
9. Attitude of medical staff toward my religion						.70	
25. Principal decision-maker for healthcare in the family							.72
26. Focus on Western (not traditional) medicine in healthcare service							.55
28. Gender difference between patient and medical staff							.51
Eigenvalues	7.76	6.89	4.29	3.99	3.04	2.54	1.71
% of variance	18.9	16.8	10.5	9.7	7.4	6.2	4.2
Cumulative variance (%)	18.9	35.7	46.2	55.9	63.3	69.5	73.7
Kaiser-Meyer-Olkin measure of sampling adequacy	0.93						
Bartlett’s test of sphericity	Approx. χ2				9879.96		
	df				820		
	*p*				<.001		

The seven factors were:

1. Hospital care and services, accounting for 18.925% of the variance, composed of 13 items on patient experience with outpatient services such as convenience of healthcare facilities, the patient-caregiver relationship, consultation waiting times, and the cost of medical care.
2. Hospital food, accounting for 16.794% of the variance, composed of seven items on quality, attitude, and cultural awareness.
3. The healthcare system, accounting for 10.464% of the variance, composed of six items on the healthcare system of South Korea such as healthcare delivery, the number of nurses per patient, and the extent of healthcare globalization.
4. Communication, accounting for 9.735% of the variance, composed of five items on verbal and nonverbal communication.
5. The healthcare facility, accounting for 7.418% of the variance, composed of four items on ward facilities that a patient may use during a hospital stay.
6. Religion, accounting for 6.202% of the variance, composed of three items on religious facilities, ceremonies, and the attitudes of medical staff.
7. Cultural values, accounting for 4.158% of the variance, composed of three items on shared ideas among patients about their own societies.

#### Validity: Hypothesis testing

The mean total score was 59.73 ± 37.17 out of a maximum of 164. A significant cultural difference (*t* = 25.716, df = 255, *p* < 0.001) was noted between how participants perceived South Korean healthcare and that of their home countries, supporting hypothesis 1 (Table 3). Table 4 lists the mean total scores and those for each individual factor, and the ANOVAs of cultural differences perceived by each language group. Significant differences between language groups (English, Chinese, Arabic, Russian, and Mongolian) were evident, supporting hypothesis 2. The total mean score was significantly higher for the Chinese than for the English and Arabic groups, and was highest for the Russian group (*p* < 0.001), who thus perceived the greatest cultural difference.

**Table 3:** Comparison of High-Score Items for Perceived Cultural Differences in Healthcare Tool of Foreign Patients

Rank	Total (*n* = 256)		Russian (*n* = 60)		Chinese (*n* = 40)	
	Items	M ± SD	Items	M ± SD	Items	M ± SD
1	Cost of medical care	2.61 ± 1.43	Cost of medical care	3.17 ± 1.27	Cost of medical care	2.78 ± 1.18
2	Food aroma	1.98 ± 1.28	Skill and competency of medical staff	2.88 ± 1.19	The globalization of healthcare service	2.16 ± 1.15
3	The globalization of healthcare service	1.93 ± 1.44	The globalization of healthcare service	2.86 ± 1.29	Focus of Western medicine (not traditional) in healthcare service	1.92 ± 1.08
4	Skill and competency of medical staff	1.91 ± 1.46	Friendliness of medical staff	2.72 ± 1.32	Spoken communication with medical staff	1.88 ± .90
5	Food taste	1.87 ± 1.24	Sanitary facilities in the hospital (e.g., toilet, baths, shower room)	2.63 ± 1.35	The religious facilities in the hospital (e.g., church, prayer room)	1.88 ± 1.20
6	Spoken communication with medical staff	1.80 ± 1.27	Caring attitude of medical staff	2.63 ± 1.23	Food appearance	1.86 ± .86
7	Convenient facilities in the hospital (e.g., bank, convenience store, café)	1.79 ± 1.34	Convenient facilities in the hospital (e.g., bank, convenience store, café)	2.54 ± 1.33	Food temperature	1.86 ± .76
8	The religious ceremonies offered at the hospital (e.g., mass, prayer)	1.79 ± 1.35	Common area in the hospital (e.g., kitchen, waiting room)	2.53 ± 1.54	The religious ceremonies offered at the hospital (e.g., mass, prayer)	1.84 ± .86
9	Understandability of information given by medical staff	1.79 ± 1.34	Protecting privacy during medical service	2.53 ± 1.34	Food taste	1.83 ± .81
10	Waiting time for consultation	1.78 ± 1.30	Understandability of information given by medical staff	2.53 ± 1.25	Food aroma	1.83 ± .77
Rank	Mongolian (*n* = 60)		English (*n* = 23)		Arabic (*n* = 73)	
	Items	M ± SD	Items	M ± SD	Items	M ± SD
1	Cost of medical care	2.95 ± 1.20	Cost of medical care	2.73 ± 1.51	Medical terms used by medical staff (e.g., brand name of medication)	1.68 ± 1.26
2	Food aroma	2.25 ± 1.27	Food temperature	2.13 ± 1.80	The religious ceremonies offered at the hospital (e.g., mass, prayer)	1.63 ± 1.52
3	Skill and competency of medical staff	2.14 ± 1.42	Food aroma	2.11 ± 1.69	Cost of medical care	1.58 ± 1.43
4	Food taste	2.00 ± 1.29	Food taste	2.00 ± 1.58	The religious facilities in the hospital (e.g., church, prayer room)	1.53 ± 1.32
5	Friendliness of medical staff	1.98 ± 1.32	The attitude of medical staff toward my food preferences	2.00 ± 1.33	Gender difference between patient and medical staff	1.48 ± 1.36
6	Food service	1.98 ± 1.22	Convenient facilities in the hospital (e.g., bank, convenience store, café)	1.95 ± 1.23	Food aroma	1.47 ± 1.23
7	Convenient facilities in the hospital (e.g., bank, convenience store, café)	1.88 ± 1.25	Sharing hospital room with other patients	1.93 ± 1.59	Waiting time for consultation	1.46 ± 1.41
8	Understandability of information given by medical staff	1.85 ± 1.31	Food service	1.90 ± 1.52	Food taste	1.46 ± 1.26
9	Waiting time for consultation	1.82 ± 1.25	The number of nurses caring for me	1.75 ± 1.57	Spoken communication with medical staff	1.43 ± 1.26
10	The globalization of healthcare service	1.80 ± 1.42	Privacy in hospital room	1.73 ± 1.53	Skill and competency of medical staff	1.38 ± 1.53

*Note. M* mean, *SD* standard deviation

**Table 4:** Comparison of Factor Scores of Tool for Measuring Cultural Differences in Healthcare Perceived by Foreign Patients

Factor number (number of items, maximum score)	English^a^ (*n* = 23)	Chinese^b^ (*n* = 40)	Arabic^c^ (*n* = 73)	Russian^d^ (*n* = 60)	Mongolian^e^ (*n* = 60)	Total (*n* = 256)	*F* (*p*)	Duncan’s test
	M ± SD	M ± SD	M ± SD	M ± SD	M ± SD	M ± SD		
1 (*n* = 13, 52)	17.48 ± 12.03	21.35 ± 9.65	14.81 ± 13.08	32.87 ± 13.53	23.18 ± 12.91	22.27 ± 14.17	18.00 (<.001)	c, a < a, b < b, e < d
2 (*n* = 7, 28)	12.30 ± 9.10	11.84 ± 4.96	7.35 ± 6.49	15.25 ± 8.25	10.47 ± 6.16	11.04 ± 7.39	10.08 (<.001)	c, e < e, b, a < b, a, d
3 (*n* = 6, 24)	7.27 ± 5.58	9.90 ± 4.27	5.59 ± 6.30	13.47 ± 7.44	8.31 ± 6.32	8.91 ± 6.90	13.74 (<.001)	c, a, e < a, e, b < d
4 (*n* = 5, 20)	6.17 ± 4.04	8.17 ± 3.89	6.89 ± 5.14	10.60 ± 5.54	7.36 ± 4.86	8.01 ± 5.11	6.20 (<.001)	a, b, c, e < d
5 (*n* = 4, 16)	5.23 ± 4.04	5.77 ± 3.01	3.40 ± 4.05	8.61 ± 5.11	5.25 ± 4.17	5.59 ± 4.58	11.64 (<.001)	c, a, e < a, e, b < d
6 (*n* = 3, 12)	2.20 ± 2.49	5.06 ± 2.55	3.23 ± 3.29	5.79 ± 3.72	3.45 ± 3.35	4.15 ± 3.44	6.28 (<.001)	a, c, e < c, e, b < b, d
7 (*n* = 3, 12)	2.33 ± 1.91	4.87 ± 2.21	3.22 ± 3.11	5.07 ± 3.72	2.89 ± 2.57	3.77 ± 3.09	7.23 (<.001)	a, e, c < b, d
Total (*n* = 41, 164)	52.91 ± 27.67	64.80 ± 25.00	43.23 ± 33.03	87.50 ± 40.72	55.12 ± 31.78	59.73 ± 37.17	16.96 (<.001)	a, c, e < e, b < d

*Note. M* mean, *SD* standard deviation. Duncan’s *post-hoc* analysis was used for unequal group variances

**Fig. 1:**
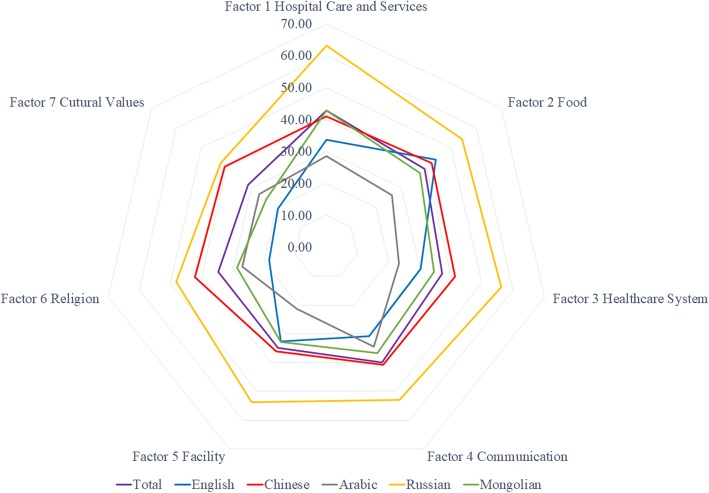
Comparison of perceived cultural differences in healthcare according to language groups

Figure 1 compares the mean scores for the various factors by language group. The cultural differences in terms of hospital care and services, food, the healthcare system, and the healthcare facility were greater than those for communication, religion, and cultural values. The Russian- and Mongolian-speaking groups showed similar patterns. The Arabic group was skewed to the left relative to the other language groups.

#### Reliability

Cronbach’s alpha was 0.96 for the complete tool, and Cronbach's alpha for individual factor ranged from 0.73 for factor 7 (cultural values) to 0.96 for factor 2 (food).

## Discussion

We developed and validated a 41-item tool measuring cultural differences perceived by foreign patients who visited South Korea for medical treatment. We explored whether cultural differences perceived by foreign patients differ  between South Korean healthcare and that of their own countries, and compared perceived cultural differences by language group. The CVIs for all items were > 0.80, and the overall CVI of the tool was 0.97, indicating that all items were relevant. EFA revealed seven significant cultural factors: hospital care and services, food, the healthcare system, communication, the healthcare facility, religion, and cultural values. Each factor contained at least three items and no item evaluated multiple factors; all components were thus unidimensional. All factor loadings were all > 0.45, indicating that all grouped items were homogeneous [39].

The seven cultural factors covered six cultural phenomena of the Giger and Davidhizar model [22], with the exception of “time”. We did not include a “time” item because we sought to measure cultural differences perceived after patients had experienced the South Korean healthcare system. However, we believe that the seven factors well-reflect the cultural phenomena of the transcultural assessment model developed by the Giger and Davidhizar to teach nursing students how to view patient culture. That is, hospital care and services, the healthcare system, and the healthcare facility all refer to environmental control, which is related to how the healthcare environment affects patient health. The food factor refers to biological variation, which is in turn related to individual uniqueness, including nutritional preferences. The communication factor of course refers to verbal communication in space (the distance between communicating/interacting individuals). The religion and cultural items refer to social organization (cultural values derived from religious affiliation, gender and sexual orientation, geography, age, and life-cycle status).

The overall Cronbach’s alpha for the tool was 0.96, and the Cronbach's alpha for all seven cultural factors were ≥ 0.70. These values are acceptable [40] and show that our tool is internally consistent.

No prior tool assessing cultural differences from the patient viewpoint is available. Babiker, Cox, and Miller [41] developed a questionnaire for use by overseas students, measuring the cultural distance between the two cultures based on their social and physical attributes. Our study is similar to the cited work in that a tool measuring differences between the two cultures was developed by dividing the cultures into measurable components. Food, religion, and communication were included in both studies. The cultural distance questionnaire for overseas students additionally examined climate, clothes, educational level, material comfort, leisure, family structure, and courtship/marriage. We additionally evaluated hospital care and services, the healthcare system, and the healthcare facility.

We used our tool to measure the extent to which foreign patients perceive cultural differences in healthcare, and which cultural factors they perceived differently. Foreign patients perceived South Korean healthcare culture as significantly different from that of their home countries. In addition, the perceived cultural differences differed among those with diverse cultural backgrounds, particularly by language group. The results support our two hypotheses and confirm that the tool measures the intended construct appropriately, i.e., it assesses cultural differences in healthcare as perceived by foreign patients. Of these differences, hospital care and services, food, the healthcare system, and the healthcare facility were more greater than personal/interpersonal factors, including communication, religion, and cultural values. The Mongolian-and Russian-speaking groups were similar, perhaps reflecting active cultural exchanges between Mongolia and Russia given their geographical proximity and shared history of bilateral relationships and co-operation. The Arabic-speaking group perceived less cultural differences than did the other patient groups, perhaps because the government and hospitals have sought to make the South Korean healthcare system as Muslim-friendly as possible; the South Korean government seeks to attract Middle Eastern patients as evidenced by the signing of a memorandum of understanding with the UAE government in 2011. Also, Muslims tend to respond more moderately (“politely”) in surveys than do other groups [42]. However, the Arabic group perceived greater cultural differences in personal/interpersonal factors.

We found that foreign patients perceived the “cost of medical care” and “skill and competency of medical staff” of the “hospital care and services” factor more differently compared to their home countries; followed by “food aroma” and “food taste” of the “food” factor and “globalization of healthcare” of the “healthcare system” factor. Our findings are similar to those of a report on foreign patients’ satisfaction surveyed by the Korean Ministry of Health and Welfare [6]. Those who participated in the foreign patients’ satisfaction survey rated South Korean medical services as more advanced than those available in their own countries, but they were not satisfied with the food or cost of treatment. The “skill and competency of medical staff” and “the globalization of healthcare” were perceived to be very different from the study respondents’ home countries. This may be related to satisfaction with the advanced South Korean medical services that they were now able to access. The “food aroma”, “food taste”, and “cost of medical care” scores, also were perceived as very different from our study respondents’ home countries. This could be due to dissatisfaction with the food and cost of treatment they receive in Korea.

The tool developed in this study will help nurses to understand the cultural needs of foreign patients, and facilitate an understanding of different cultures. The strength of the study was that we obtained data from 256 patients from various countries, with this sample showing cultural  differences across the different language groups. Nurses can use the tool to assess patients from different cultures and respect cultural differences when caring for foreign patients. Nurses can distribute questionnaires to foreign patients, study the responses, and adjust their care accordingly. Patient-centered care and evidence-based nursing care will thus be achievable.

Our study had certain limitations. Although we measured perceived cultural differences, we did not measure the direction of such differences. In future study, the tool should include descriptive phrases to reveal the direction of differences (positive or negative), and thus to determine how such differences impact patient care. Second, participants were recruited from only three tertiary hospitals in Seoul, South Korea, which is the most advanced city in the country. Thus, additional data should be obtained from patients in other healthcare institutions in other parts of South Korea. Third, although we performed EFA and used Cronbach’s alpha to confirm the validity and reliability of the tool, further testing is required.

## Conclusions

We developed a tool that measures cultural differences in healthcare perceived by foreign patients in South Korea. Healthcare providers can use the tool to measure the extent of such differences in particular healthcare domains. Nurses can use the tool to understand foreign patients and render their care more culturally competent.

## Abbreviations

ANOVA: Analysis of variance
CVI: Content validity index
EFA: Exploratory factor analysis
KMO: Kaiser-Meyer-Olkin
OECD: Organization for Economic Cooperation and Development
SD: Standard Deviation
UAE: United Arab Emirates
USA: United States of America
